# Closing the Loop: Modelling of Heart Failure Progression from Health to End-Stage Using a Meta-Analysis of Left Ventricular Pressure-Volume Loops

**DOI:** 10.1371/journal.pone.0114153

**Published:** 2014-12-05

**Authors:** David R. Warriner, Alistair G. Brown, Susheel Varma, Paul J. Sheridan, Patricia Lawford, David R. Hose, Abdallah Al-Mohammad, Yubing Shi

**Affiliations:** 1 Medical Physics Group, Department of Cardiovascular Science, University of Sheffield, Sheffield, S10 2TN, United Kingdom; 2 Department of Cardiology, Northern General Hospital, Sheffield Teaching Hospitals, Sheffield, S5 7AU, United Kingdom; San Diego State University, United States of America

## Abstract

**Introduction:**

The American Heart Association (AHA)/American College of Cardiology (ACC) guidelines for the classification of heart failure (HF) are descriptive but lack precise and objective measures which would assist in categorising such patients. Our aim was two fold, firstly to demonstrate quantitatively the progression of HF through each stage using a meta-analysis of existing left ventricular (LV) pressure-volume (PV) loop data and secondly use the LV PV loop data to create stage specific HF models.

**Methods and Results:**

A literature search yielded 31 papers with PV data, representing over 200 patients in different stages of HF. The raw pressure and volume data were extracted from the papers using a digitising software package and the means were calculated. The data demonstrated that, as HF progressed, stroke volume (SV), ejection fraction (EF%) decreased while LV volumes increased. A 2-element lumped parameter model was employed to model the mean loops and the error was calculated between the loops, demonstrating close fit between the loops. The only parameter that was consistently and statistically different across all the stages was the elastance (Emax).

**Conclusions:**

For the first time, the authors have created a visual and quantitative representation of the AHA/ACC stages of LVSD-HF, from normal to end-stage. The study demonstrates that robust, load-independent and reproducible parameters, such as elastance, can be used to categorise and model HF, complementing the existing classification. The modelled PV loops establish previously unknown physiological parameters for each AHA/ACC stage of LVSD-HF, such as LV elastance and highlight that it this parameter alone, in lumped parameter models, that determines the severity of HF. Such information will enable cardiovascular modellers with an interest in HF, to create more accurate models of the heart as it fails.

## Introduction

To model LV performance, quantitative data, such as pressure and volume, is vital to ensure that any model is an accurate representation of reality. As such, a computational model of the LV cannot be built on single parameters alone such as left ventricular end-diastolic volume (LVEDV), subjective symptoms such as dyspnoea, nor surrogate markers such as natriuretic peptides (NP's) but direct measures of the system being modelled, both anatomical e.g. volume and physiological e.g. pressure.

Previous attempts to model the HF, and the effect of potential therapies, have applied hypothetical haemodynamic states according to symptomatic New York Heart Association (NYHA) class rather than actual patient data, from individuals or populations. The NYHA class, whilst useful clinically, correlates poorly with even non-invasive measures of LV performance [1], [2]. Current computational models of HF, regardless of complexity, choose arbitrary parameters for the LV such as reducing contractility by 50% or boundary conditions such as resistance and compliance from healthy populations. Clearly, it is not just the “pump” that fails during HF, but also the vasculature, among other systems, and each may augment the decline of the other. The authors aimed to provide specific LV performance and systemic vascular data on a population basis, to track the progression from a healthy to a failing heart. In doing so, for the first time, give the modelling community access to disease and severity specific variables derived from real patients, to enable the creation of more accurate models.

In 2001, the joint American Heart Association (AHA)/American College of Cardiology (ACC) guidelines categorised for the first time HF and its progression in terms of pathophysiology (see table 1). This was intended to “complement” the pre-existing NYHA functional classification and the development of HF, from risk e.g. hypertension (Stage A) to end-stage e.g. requiring transplant (Stage D) [3]. Whilst the guidelines have subsequently been updated, the classification remains qualitative and is often misunderstood [4], [5]. The addition of quantitative measures for each stage could be used to more accurately chart the pathophysiology and enable the development HF models based on objective parameters. Such measures may also improve risk stratification and predict response to clinical interventions. To date, attempts to categorize patients into individual AHA/ACC stages have focussed on indirect measures, such as NP's, which nonetheless improve prognostication [6].

**Table 1:** The American Heart Association/American College of Cardiology Heart Failure classification From Jessup et al. (2009) [4].

Stage	Description	Examples
A	Risk of heart failure but without structural heart disease	e.g. Hypertension
B	Structural heart disease without signs or symptoms	e.g. Previous myocardial infarction
C	Structural heart disease with prior or current symptoms	e.g. Patients requiring routine drugs
D	Refractory heart failure requiring specialist intervention	e.g. Heart transplant

The authors wished to define quantitatively each AHA/ACC stage of HF and so for the first time define the risk and the onset and progression of left ventricular systolic dysfunction heart failure (LVSD-HF) according to objective changes in left ventricular physical properties represented by the left ventricular pressure-volume loops. The choice of the pressure-volume (PV) loop is based on its direct description of the performance of the LV in real-time and for reasons mentioned above, quantifying LVSD-HF by other parameters such as LV volume or symptoms is insufficient for modelling purposes.

For this study, we concentrated on the chronic LVSD-HF population based on both pragmatic approach, and a more therapeutic consideration since only patients with LVSD-HF have interventions that are associated with significant impact on morbidity and mortality. There have been many studies looking at LV PV loops in patients with heart disease, but this is the first time they have all been collated, compared and modelling parameters derived. For comparision and completeness, a healthy normal group (stage O) was created also, not contained in the AHA/ACC guidelines,

## Methods

### PV loops

**Figure 1:**
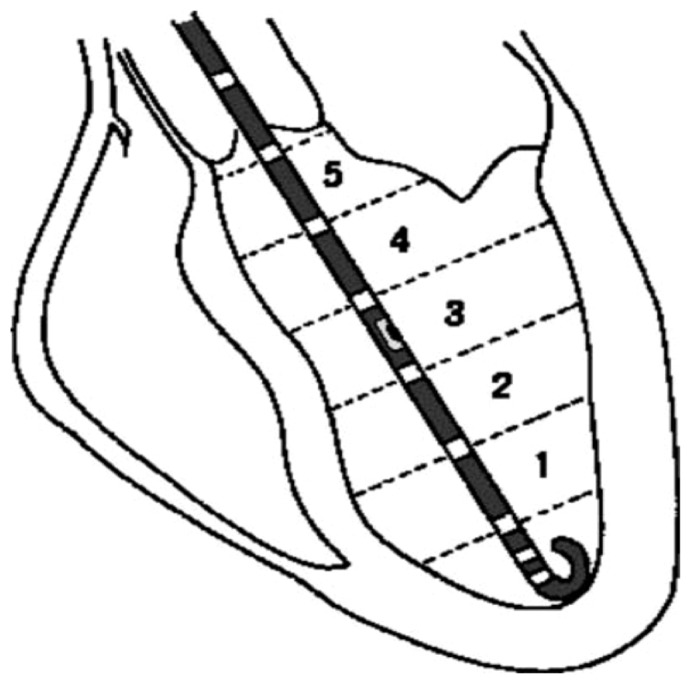
Combined pressure-volume catheter (in black) positioned in the left ventricular cavity. The micromanometer can be seen at level 3 and the 6 electrodes (white markers) mapping the volume of each of the 5 segments (reproduced with permission, from Steendijk et al. (2004)).

The methodology for PV loop acquisition has been described in detail previously [7], [8]. Briefly, a specialised catheter is inserted via the femoral artery to the apex of the LV cavity under fluoroscopy (see figure 1). Real-time measurement of pressure is performed using a micro manometer on the catheter, and of volume, using the conductance method. The conductance method refers to the usage of linearly placed electrodes on the catheter, each measuring segmental volumes, this utilises excitation and recording electrodes, the former generate an electric field, and the latter measure a change in voltage proportional to resistance. A mathematical formula is then used to calculate the total volume of the LV cavity, which takes into account the distance between the electrodes and the blood pool resistivity.

**Figure 2:**
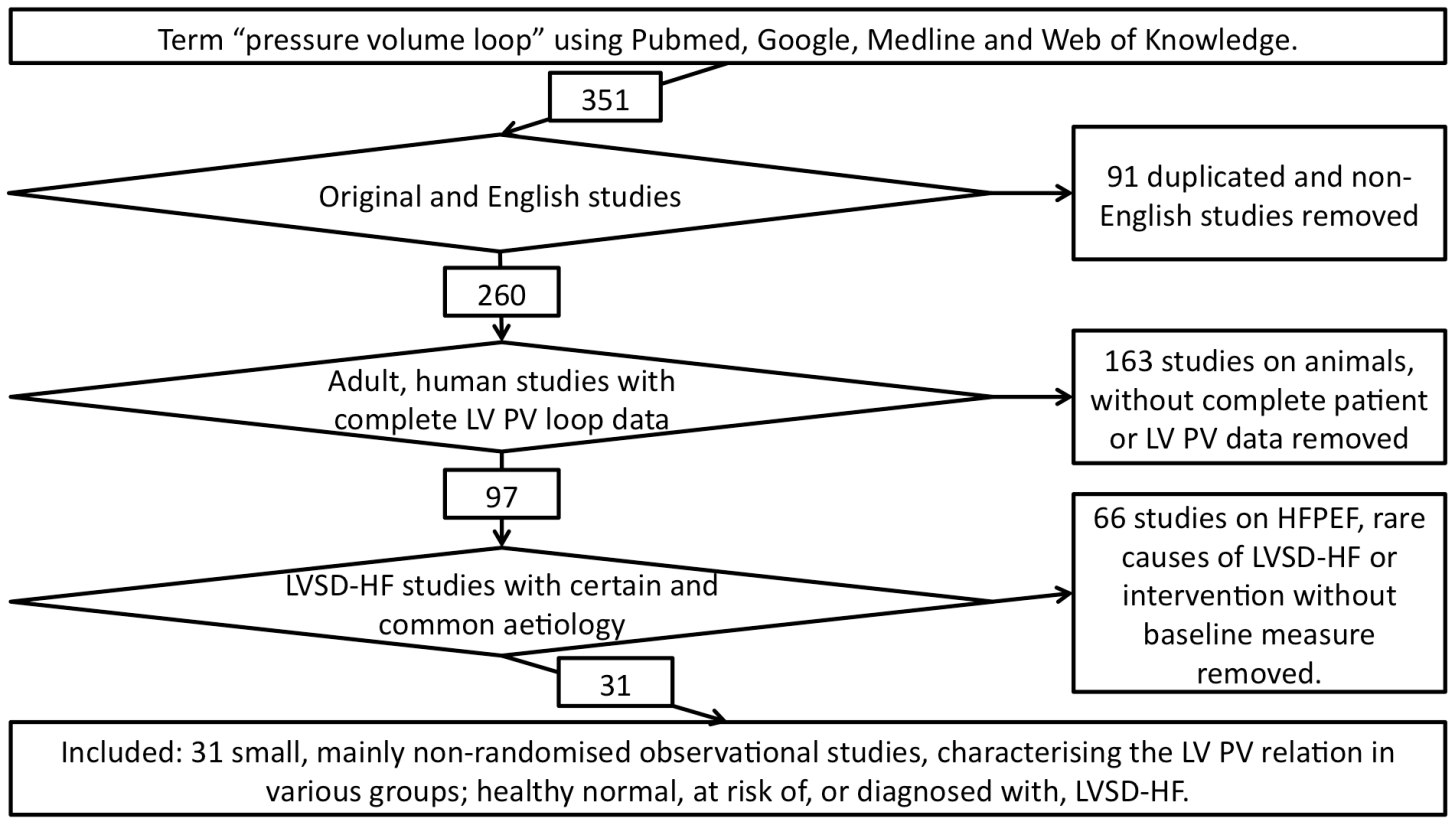
Flow diagram demonstrating inclusion and exclusion criteria for left ventricular pressure-volume loop studies. HFPEF  =  heart failure with preserved ejection fraction, LV  =  left ventricle, LVSD  =  left ventricular systolic dysfunction, PV  =  pressure volume,

An online literature search of Pubmed, Web of Knowledge, Medline and Google, using the search term “pressure volume loop” was conducted and the references were studied to check that they met criteria to be included in the final analysis (see figure 2).

### Inclusion criteria

Studies with complete LV PV loops in adult humans in English.

(and) Studies representing any AHA/ACC stage including healthy normal subjects.

(and) HF, if present, due to chronic LVSD-HF only.

### Exclusion criteria

Diastolic HF or HFPEF.

HF secondary to an uncommon cause such as Chagas' disease.

An unclear past medical, symptom or drug history, meaning HF stage was vague.

No pictorial representation of an entire loop, such as a diastolic limb only.

Acquisition during experimental treatment only without a baseline measure

Of the papers identified, 97 were potentially useable, but only 31 met the inclusion, and not the exclusion, criteria and so were included in the final analysis, accounting for 203 patients [9]–[38].

**Figure 3:**
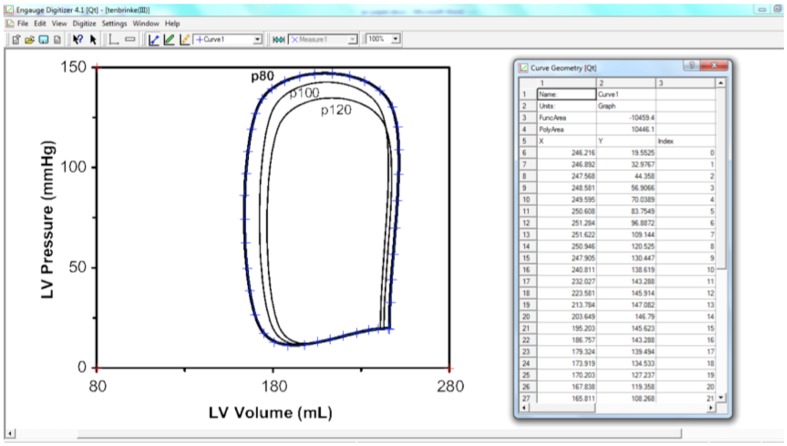
Screenshot from Engauge - on the left, the pressure volume loop is seen, with red cross hairs denoting X and Y axes, and blue crosshairs which correspond with the numerical values of pressure and volume seen in the table on the right. LV  =  left ventricle.

Engauge digitizing software (http://digitizer.sourceforge.net/) was then used to upload the graphical PV loop images from the original studies and convert them into numerical data, as seen in figure 3 [39]. This freeware allows users to upload a graphical image, such as a PV loop, in variety of formats such as a Joint Photographic Experts Group (JPEG) file, and convert a pictorial image into numerical data. After loading the file, the parameters of the axes (red crosshairs) were chosen, with X corresponding to 80–280 ml and Y 0–150 mmHg respectively. The PV loop is then digitised automatically (blue crosshairs), turning the PV loop picture into a series of individual pressure and volume data points, 10 points per limb of the curve, giving a total of 40 data points for each PV loop. The LV PV loops from each LVSD-HF stage were converted into digital values and these were then tabulated and the mean for each calculated.

### Modelling the PV loops

Various computational models exist in the engineering literature of the cardiovascular system, from simple lumped parameter to more complex 3 dimensional models. The purpose of the lumped parameter models is to describe the changes in pressure, volume and flow that occur over the cardiac cycle as a function of cardiac performance and systemic afterload. This paper presents the numerical values of the four parameters in the simplest possible representation of the heart and systemic circulation; LV elastance (maximum and minimum), total peripheral resistance and systemic vascular compliance, the components of which are described below. The progression of LVSD-HF is thus expressed in terms of the evolution of these four parameters. Furthermore it is suggested that the values of the components that represent the systemic afterload might be used to determine appropriate boundary conditions for the modeller who is interested in using such representations for complex 3D models of the left ventricle, which still rely on a specific afterload for the LV to “push” against.

A lumped parameter model represents complex systems such as the cardiovascular system as a hydro-electrical analogue. In particular, the Windkessel model (meaning “air chamber” in German) contains a two element afterload, a capacitor representing the elastic property of the large arteries which determines systemic vascular compliance (C) and a resistor representing the frictional loss in the smaller vessels e.g. arterioles which determine the total peripheral resistance (R). The LV is represented by a variable elastance (E) model, where by the LV pressure is a function of the LV elastance and the change in LV volume from its resting state, in this model E is represented by 2 values, Emax being representative of peak systolic LV contractility and Emin being representative of end diastolic LV stiffness. Using software such as OpenCell, (http://www.cellml.org/tools/opencell) such models can be run and the input variables of E, R and C manipulated, for example, to model hypertension one could reduce the compliance and increase the resistance of the vasculature, whilst leaving the value of E unaltered. The resulting outputs of LV pressure and volume can be exported to a database, extracted from one cardiac cycle and then converted into a PV loop.

**Figure 4:**
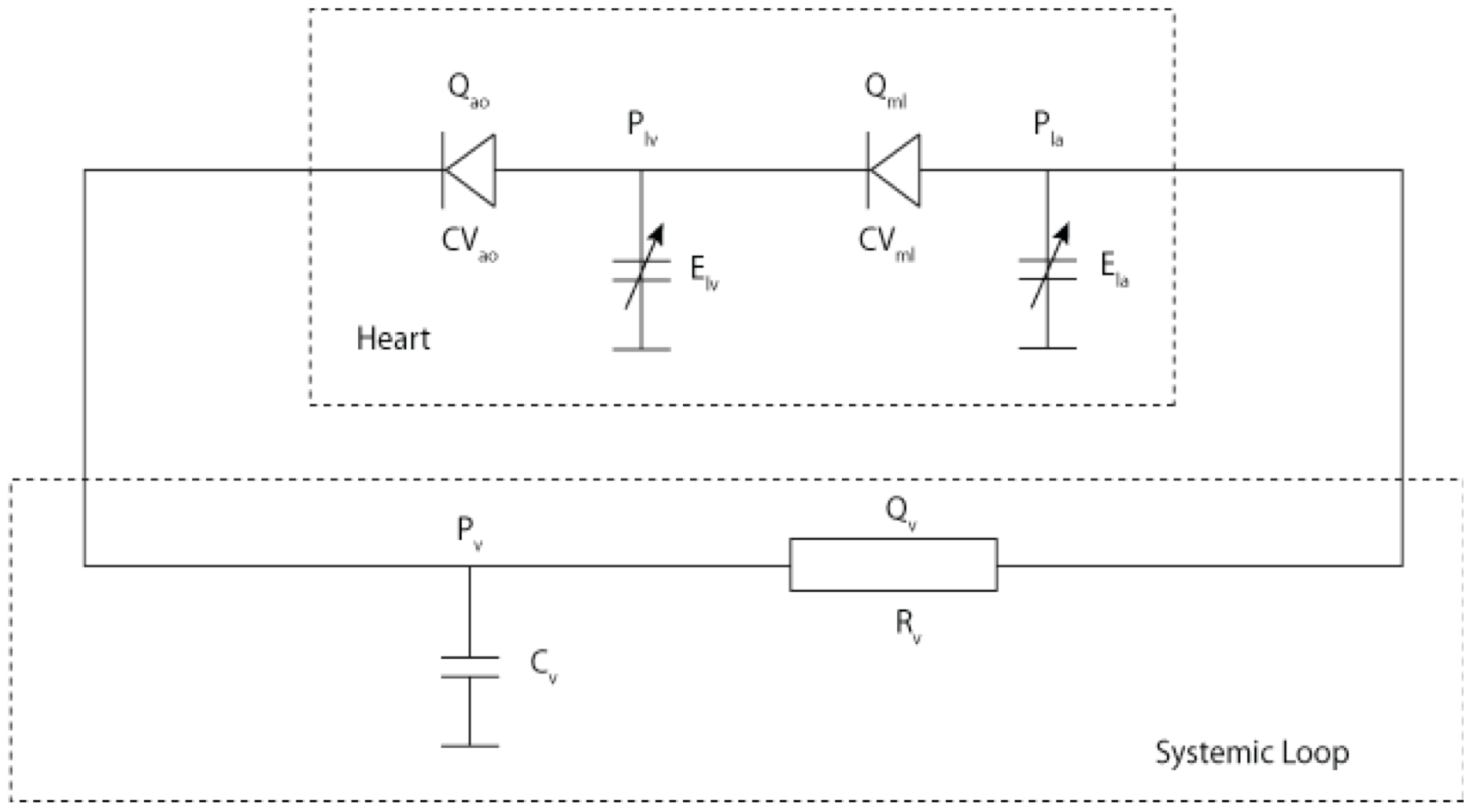
Schematic diagram of Zero-D model of the cardiovascular system, with the heart comprised of variable capacitors representing elastance of the LA (E_LA_) and LV (E_LV_) and the aortic (_ao_) and mitral valves (_mi_) by diodes. The systemic loop is comprised of a systemic arterial compliance represented by a capacitor (C_V_) and total peripheral resistance by a resistor (R_V_).

For this study, a lumped parameter model with a variable elastance LV and 2 element (R and C) Windkessel afterload was chosen to model the LV in LVSD-HF [40]. It was chosen due to its elegance in representing the cardiovascular system, simplicity in manipulation, low computational demands and experience within the research group. This was downloaded from the CellML (http://www.cellml.org/) model repository, which is a free to access store of computer based mathematical models, and run using OpenCell, an open source platform for working with CellML models, see figure 4 [41]. In this model the left atrium (E_LA_) and left ventricle (E_LV_) are represented by variable capacitors to model the pumping action of the left side of the heart, the mitral (_mi_) and aortic (_ao_) valves are represented by diodes to model unidirectional flow, the total peripheral resistance by a resistor, systemic vascular compliance by a capacitor and blood vessels by wire allowing for flow of electrons, representing the flow of blood.

To model the mean AHA/ACC PV loops, the Matlab (Mathworks, MA, USA) optimisation toolbox was used to find the combination of parameters that best fitted the data. This toolbox enabled running the lumped parameter model as an iterative process, whilst varying each variable (Emax, Emin, R and C) until the best-fit model for the patient derived mean LV PV loop for each stage was created. The resulting model PV loop data was exported to a spreadsheet and compared against the mean loops (see below).

### Statistics

Statistical analysis was carried out using Microsoft Excel 2010 software (Microsoft, CA, USA). Parametric data is given as mean (± SD). Comparison of data between LVSD-HF stages using an unpaired 2 tailed Students t-test and *p* values of <0.05 were considered significant.

## Results

### Mean PV loops

The majority of the patients making up each category are males in their late fifties (see table 2). Some AHA/ACC LVSD-HF stage groups have more patients than others and group A is dominated by ischaemic heart disease (IHD), rather than other risk factors such as obesity or diabetes. However, there is a balanced distribution of LVSD-HF aetiologies in both groups C and D, with both ischaemic and idiopathic dilated cardiomyopathy (DCM) accounting for approximately 50% each.

**Table 2:** Demographic information on the patients comprising the left ventricular pressure volume loops.

AHA/ACC HF Stage			O	A	B	C	D
Demographics	Number of patients	Total population	20	144	88	62	129
		Patients with loops	2	65	6	42	92
	Gender	% male	75	65	77	88	84
	Age	mean	29	56	59	60	58
Aetiology	HTN	%		7			
	IHD	%		93			
	MI	%			100		
	Ischaemic DCM	%				50	54
	Idiopathic DCM	%				50	46

HTN – hypertension, IHD – ischaemic heart disease, MI – myocardial infarction, DCM – dilated cardiomyopathy.

**Figure 5:**
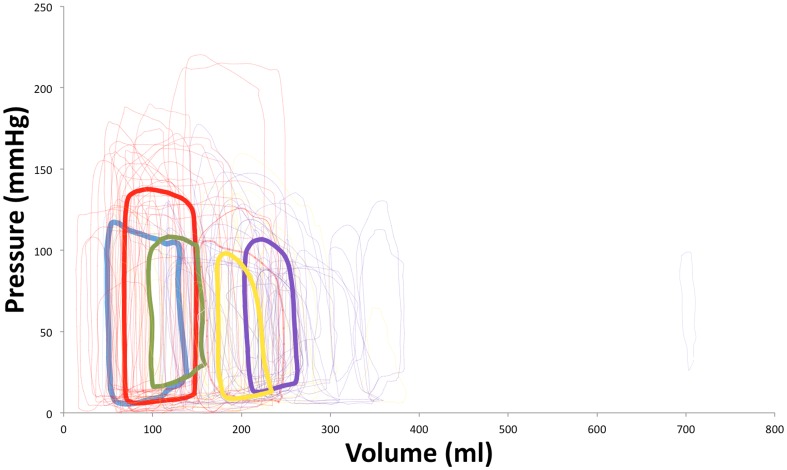
Graph showing the progression of HF by individual PV (thin, pale lines) and averaged (thick, dark lines) loops for all the American Heart Association (AHA)/American College of Cardiology (ACC) heart failure (HF) stages including stage O (blue), A (red), B (green), C (yellow) and D (purple).

**Figure 6:**
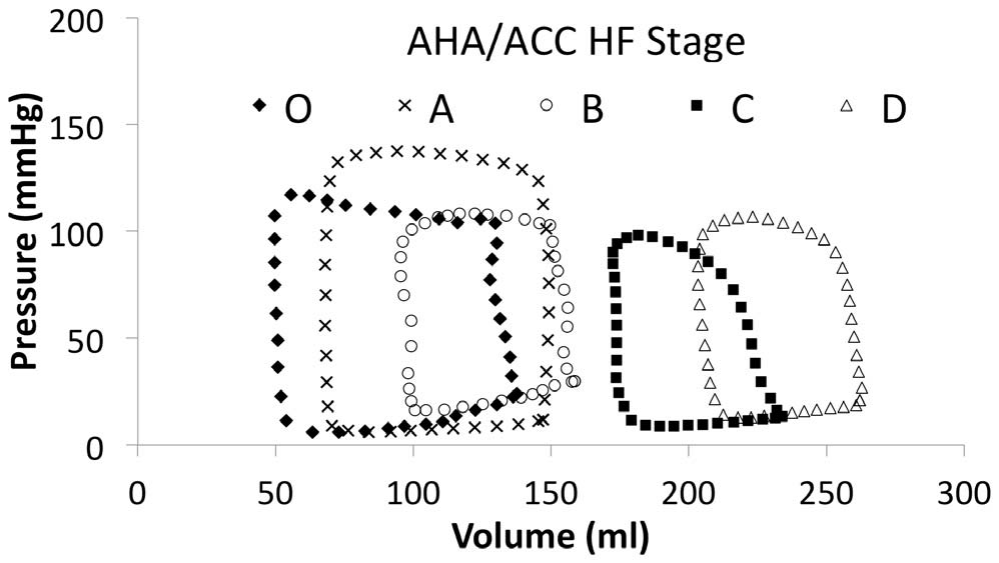
Graph showing the progression of HF by mean PV loops for all the American Heart Association (AHA)/American College of Cardiology (ACC) heart failure (HF) stages including stage O (solid black diamonds), A (black crosses), B (white circles), C (solid black squares) and D (white triangles).

Figure 5 demonstrates all of the individual patient loops together with the mean loops from each stage. As one can see from figure 6, there is a conformational difference not only between all stages O-D but also between those asymptomatic or at risk groups A-B and those in symptomatic LVSD-HF groups C-D. Table 3 shows that as one progresses from normal LV function to symptomatic LVSD-HF due to left ventricular systolic dysfunction: the LV volumes and diastolic pressures rise, and the stroke volume (SV), ejection fraction (EF%) and systolic pressure fall. Furthermore, the maximal elastance of the LV falls, as the disease progresses.

**Table 3:** Mean left ventricle parameters for each stage of heart failure (with standard deviation in brackets).

AHA/ACC HF Stage		O	A	B	C	D
LV parameters	LVESV (ml)	48 (21)	66 (52)	93 (48)	166 (61)	210 (96)
	LVEDV (ml)	138 (7)	154 (33)	161 (49)	237 (62)	273 (98)
	SV (ml)	89 (14)	88 (29)	68 (23)	71 (21)	63 (30)
	EF (%)	0.65 (0.01)	0.57 (0.10)	0.45 (0.16)	0.32 (0.10)	0.25 (0.10)
	Elastance (mmHg/ml)	2.23 (0.26)	2.27 (0.29)	1.32 (0.73)	0.63 (0.36)	0.55 (0.23)
	Stiffness (mmHg/ml)	0.17 (0.01)	0.09 (0.06)	0.10 (0.03)	0.06 (0.04)	0.08 (0.04)

LV  =  left ventricle, LVESV  =  left ventricular end systolic volume, LVEDV  =  left ventricular end diastolic volume, EF  =  ejection fraction.

**Figure 7:**
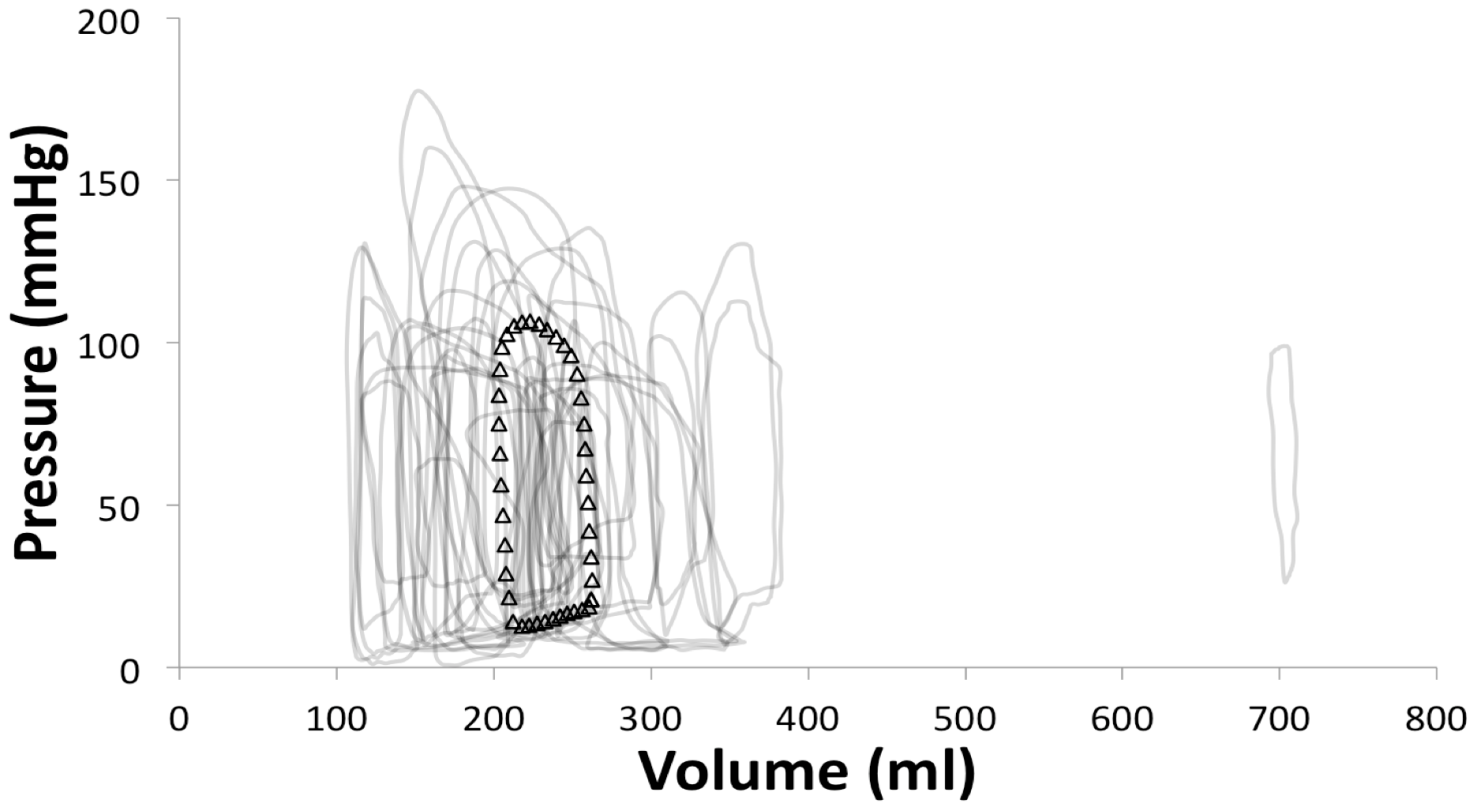
Graph showing mean pressure volume loop for American Heart Association (AHA)/American College of Cardiology (ACC) heart failure (HF) stage D (white triangles) and the spread of the raw loops sourced from the literature (grey lines).

**Figure 8:**
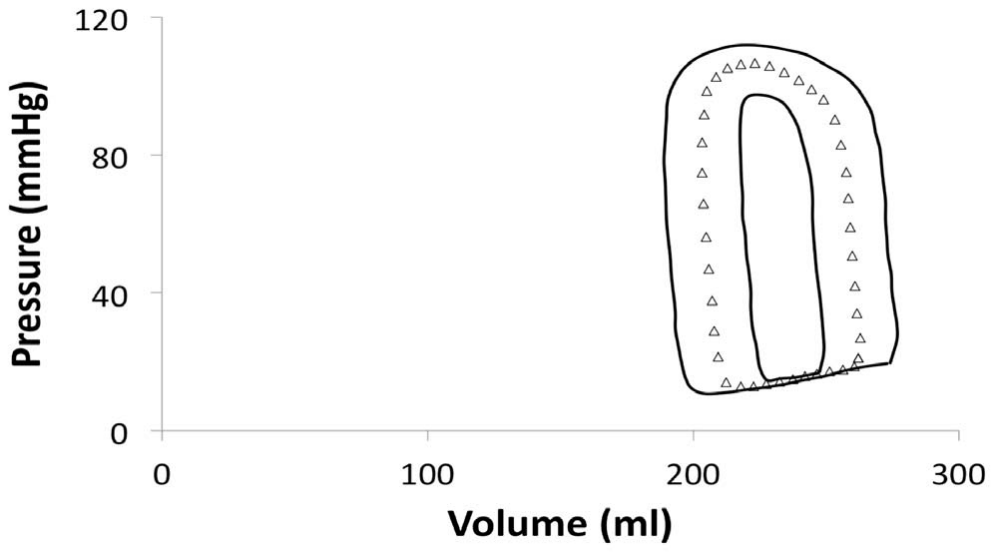
Graph showing mean pressure volume loop for each American Heart Association (AHA)/American College of Cardiology (ACC) heart failure (HF) stage (shown as white triangles) and standard error (shown black lines).

For clarity, we have just shown the raw and mean loops from one stage, AHA/ACC D (figure 7). It is evident that even within AHA/ACC stages there is individual variation, both in terms of LV pressure and volume. Figure 8 demonstrates the standard error for each of the PV points derived from patients in this stage, reflecting this large spread. Table 4 demonstrates, that there is no statistically significance differences between stages O and A or B, but together they are significantly different from stages C and D in all variables, other than other than minimal elastance. There are no significant differences between stages C and D.

**Table 4:** Statistical comparison of parameters from the patient data for each American Heart Association/American College of Cardiology heart failure stage, using 2-tailed Student's T-Test.

AHA/ACC HF Stage	Parameter	Unit	O	A	B	C	D
O	LVEDV	(ml)		*p* = 0.31	*p* = 0.27	*p*<0.05	*p*<0.05
	LVESV	(ml)		*p* = 0.22	*p* = 0.13	*p*<0.01	*p*<0.05
	SV	(ml)		*p* = 0.49	*p* = 0.13	*p* = 0.11	*p*<0.001
	EF	(%)		*p* = 0.19	*p*<0.05	*p*<0.001	*p*<0.001
	Emax	(mmHg/ml)		*p* = 0.48	*p* = 0.05	*p*<0.001	*p*<0.001
	Emin	(mmHg/ml)		*p* = 0.29	*p* = 0.19	*p* = 0.08	*p* = 0.39
A	LVEDV	(ml)			*p* = 0.37	*p<0.001*	*p*<0.001
	LVESV	(ml)			*p* = 0.05	*p*<0.001	*p*<0.001
	SV	(ml)			*p*<0.05	*p*<0.01	*p*<0.001
	EF	(%)			*p*<0.01	*p*<0.001	*p*<0.001
	Emax	(mmHg/ml)			*p*<0.05	*p*<0.001	*p*<0.001
	Emin	(mmHg/ml)			*p* = 0.47	*p*<0.001	*p* = 0.21
B	LVEDV	(ml)				*p*<0.01	*p*<0.01
	LVESV	(ml)				*p*<0.01	*p*<0.001
	SV	(ml)				*p* = 0.39	*p*<0.001
	EF	(%)				*p*<0.01	*p*<0.001
	Emax	(mmHg/ml)				*p*<0.01	*p*<0.001
	Emin	(mmHg/ml)				*p*<0.001	*p* = 0.29
C	LVEDV	(ml)					*p* = 0.09
	LVESV	(ml)					*p* = 0.08
	SV	(ml)					*p* = 0.40
	EF	(%)					p<0.05
	Emax	(mmHg/ml)					*p* = 0.38
	Emin	(mmHg/ml)					*p = 0.18*

AHA  =  American Heart Association, ACC  =  American College of Cardiology EF  =  ejection fraction, LV  =  left ventricle, LVESV  =  left ventricular end systolic volume, LVEDV  =  left ventricular end diastolic volume, SV  =  stroke volume.

### Modelled PV loops

**Figure 9:**
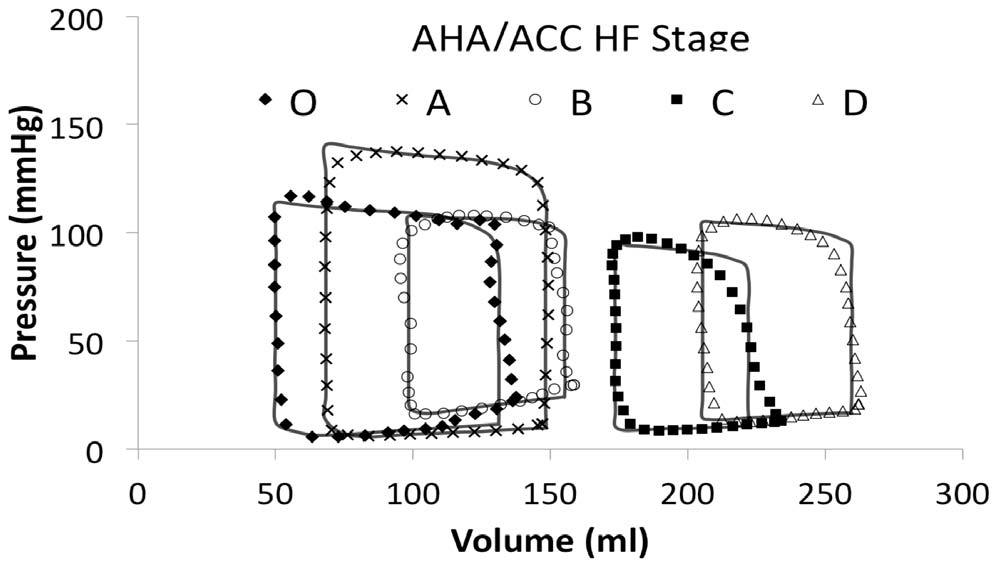
Graph showing the progression of heart failure by mean pressure volume loop for all American Heart Association (AHA)/American College of Cardiology (ACC) heart failure stages from O-D (various markers) along with the modelled loops (solid black lines).

For the modelled PV loops (see figure 9) there is a more accurate fit for the loops representing the earlier AHA/ACC LVSD-HF stages, which reduces as the LV contraction deteriorates and the stage progresses. Table 5, shows how the modelled LV elastance falls from a normal LV to end-stage HF due to LVSD; the volume of the LV increases but yet the resistance and compliance of the systemic vasculature remain unchanged.

**Table 5:** Lumped parameter model variables and calculated error for each stage.

Parameter	Unit	AHA/ACC HF Stage				
		O	A	B	C	D
Emax	(mmHg/ml)	2.50	2.20	1.14	0.55	0.52
Emin	(mmHg/ml)	0.08	0.06	0.15	0.04	0.06
LV Volume	(ml)	468	522	771	579	726
Resistance	(mmHg•s/ml)	1.15	1.51	1.50	1.65	1.58
Compliance	(ml/mmHg)	3.19	2.90	5.34	3.87	4.33
Percentage error	(%)	7.5	3.0	7.5	15.0	10.6

Table 6 demonstrates, that there is no statistically significance difference between model parameters between stages O and A or B, but they are significantly different from stages C and D in Emax only. There were no significant differences between stages C and D.

**Table 6:** Statistical comparison of the lumped parameter variables for each American Heart Association/American College of Cardiology heart failure stage model, using 2-tailed Student's T-test.

AHA/ACC HF Stage	Parameter	Unit	O	A	B	C	D
O	Emax	(mmhg/ml)		*p* = 0.76	*p* = 0.81	*p*<0.001	*p*<0.001
	Emin	(mmhg/ml)		*p* = 0.71	*p* = 0.65	*p* = 0.98	*p* = 0.77
	LV Volume	(ml)		*p* = 0.76	*p* = 0.81	*p* = 0.17	*p*<0.001
	Resistance	(mmhg•s/ml)		*p* = 0.06	*p* = 0.25	*p* = 0.48	*p* = 0.37
	Capacitance	(ml/mmhg)		*p* = 0.67	*p* = 0.93	*p* = 0.71	*p* = 0.80
A	Emax	(mmhg/ml)			*p* = 0.63	*p*<0.001	*p*<0.001
	Emin	(mmhg/ml)			*p* = 0.06	*p*<0.05	*p*<0.001
	LV Volume	(ml)			*p* = 0.27	*p*<0.05	*p* = 0.53
	Resistance	(mmhg•s/ml)			*p* = 0.36	*p* = 0.21	*p* = 0.08
	Capacitance	(ml/mmhg)			*p* = 0.41	*p* = 0.06	*p* = 0.07
B	Emax	(mmhg/ml)				*p*<0.01	*p*<0.001
	Emin	(mmhg/ml)				*p* = 0.16	*p*<0.001
	LV Volume	(ml)				*p* = 0.88	*p* = 0.35
	Resistance	(mmhg•s/ml)				*p* = 0.88	*p* = 0.35
	Capacitance	(ml/mmhg)				*p* = 0.63	*p* = 0.35
C	Emax	(mmhg/ml)					*p* = 0.43
	Emin	(mmhg/ml)					*p* = 0.25
	LV Volume	(ml)					*p* = 0.25
	Resistance	(mmhg•s/ml)					*p*<0.05
	Capacitance	(ml/mmhg)					*p* = 0.9

**Figure 10:**
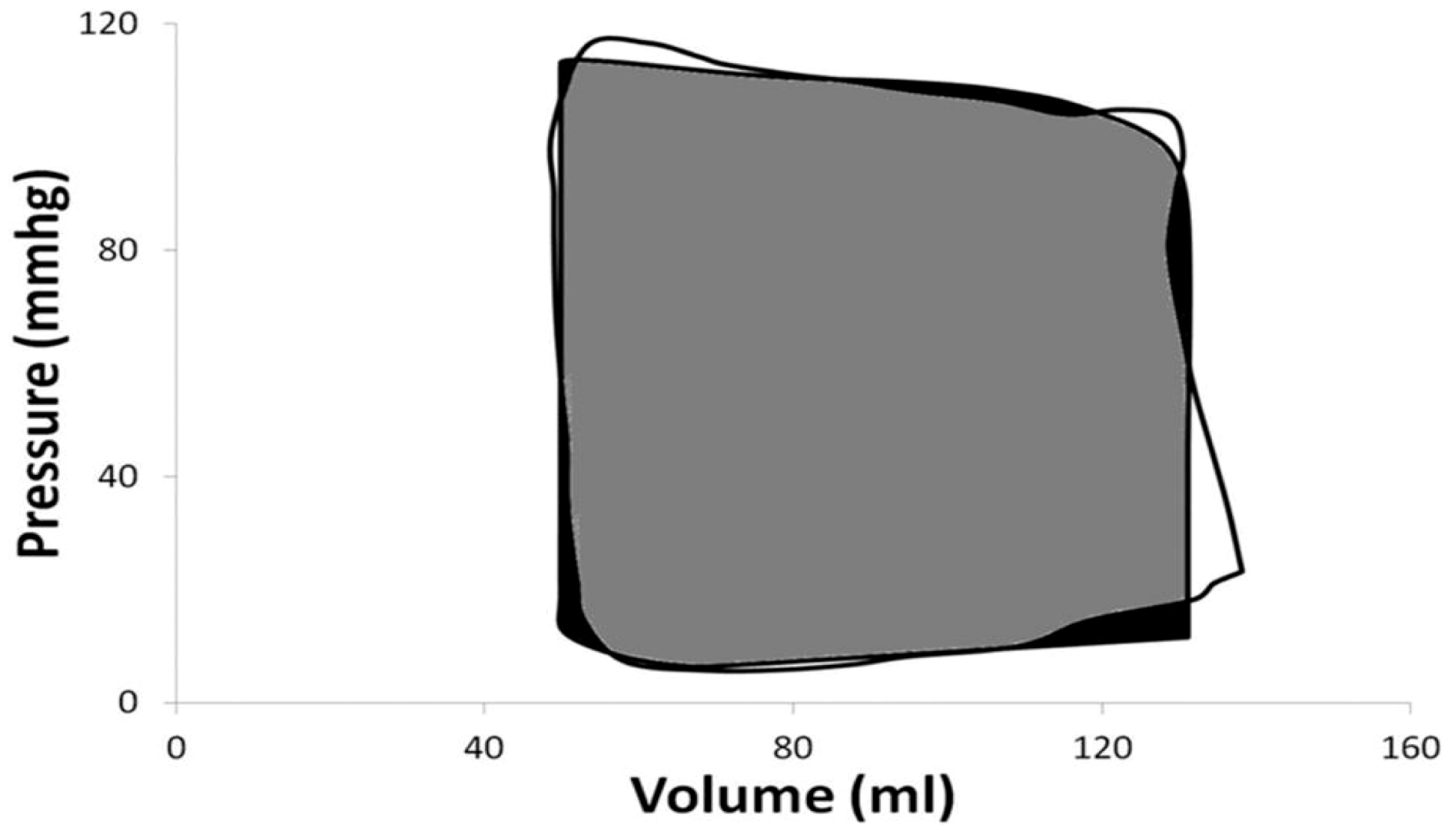
Graph demonstrating the PV area comparison of the mean loop for American Heart Association/American College of Cardiology heart failure stage O (shown in white), the modelled loop (shown in black) and the intercepting area (shown in grey).

Using Matlab (Mathworks, MA, USA), we compared the area error for the modelled loops to the mean, to give a measurement of accuracy (see table 5 and figure 10). This method compared the area occupied by the mean PV loop derived from the patient (white) data against the mean PV loop created from the lumped parameter model (black) to give an overlapping area (grey). Because the modelled loops are based on mean data, modelling the size, rather than the shape of the PV loops is important. Thus we compared the overlapping area of the mean and modelled loops as a measure of closeness of fit against that area which did not overlap. The area, which was not overlapping e.g. the error, was calculated as a percentage of the total, giving an overall mean error for all stages of less than 10%. Comparing the mean and modelled PV loop data statistically; there were no significant differences at any stage (not shown).

## Discussion

This is the first meta-analysis of all of the existing literature on PV loops in LVSD-HF, stratified by AHA/ACC stage. This study demonstrated that the pressure-volume data in the literature supports the existing theoretical ACC/AHA physiological paradigm for LVSD-HF, whereby as the left ventricle fails, it dilates. Thus, the contractile force is impaired and the volume it ejects with each beat is reduced. This study showed quantitatively the changes between each AHA/ACC stage. As the AHA/ACC stages are descriptive and qualitative, particularly stages C and D, it is remarkable how the stages could be delineated quantitatively in this study. Indeed, it demonstrates the strength of the EF, although typically used in echocardiography or cardiac magnetic resonance, as it is the only variable in the patient data, that is statistically and significantly different across all the stages, other than between O and A.

For the first time, a LPM has been used to model all the existing PV loop data across the entire spectrum of heart disease, from healthy normal individuals to patients with end-stage LVSD-HF. The model, whilst simple, performed well, with a mean error of less than 10% and gives insight into how the heart, and the systemic vasculature fail.

The lumped parameter model is true in physiological sense. Predictably, the pump function of the left ventricle deteriorates as LVSD-HF progresses, the left ventricular chamber dilates, the afterload and plasma volume increase, which will affect both R and C. The pressure-volume loops showed each stage as expected, thus: Stage O is indeed healthy, with normal left ventricular parameters that we would expect from a disease-free population. Patients in Stage A, the vast majority of whom have IHD, the systolic pressure rises reflecting increased afterload and the EF% and SV fall. However, all these parameters are still within normal limits. Following an ischaemic insult to the myocardium in Stage B, there is a rise in LVEDP reflecting increased stiffness, a fall in systolic pressure due to impaired contractile force and corresponding reduction in both the ejection fraction and the stroke volume. What is somewhat surprising is that whilst there is an increase in volume from stage C to D, both the systolic and diastolic pressures rise. This is not necessarily surprising as the process is not a simple mechanical process but it reflects the contribution of the compensatory mechanisms driven by the sympathetic drive and by the endocrine responses driven by the rennin-angiotensin-aldosterone system as well as the sympathetic system.

The patients in stages A-B are asymptomatic from the LVSD-HF viewpoint, this is despite an increase of R and C, and decrease in LV Emax, by 60% (relative to Stage O). However, the remaining contractile force is sufficient to meet the demands of the body. The difference between the asymptomatic patients with structural heart disease (Stage B) and those with symptoms of (Stage C) is the reduction in LV Emax by a further 50%. The difference between symptomatic and refractory LVSD-HF is a further modest (13%) reduction in Emax (which translates into an absolute fall of 75% compared to Stage O). This is an important observation that illustrates the delicate tipping point between physiological compensation and decompensation. Indeed, as seen statistically, stage C and D were not significantly different from one another, but they were on almost every single variable compared to stages O-B. This information, whilst logical and perhaps inferred, was previously unknown. Also, there was no significant difference between any of the groups in Emin, representing LV end-diastolic stiffness, as one might expect, as this was a study investigating LVSD-HF, not HFPEF, although diastolic dysfunction can co-exist with systolic.

Importantly, the creation of the lumped parameter model variables for the various LVSD-HF stages (see table 4), will satisfy both the academic and clinical communities. From simple lumped parameter to 3D models of the LV, all use pre-defined boundary conditions, such as elastance, resistance and capacitance, regardless of their complexity. From a clinical perspective, a model of the failing heart, should utilise as much patient derived data as possible, not simply use arbitrary measures. From a modelling perspective, a model must be based on robust, repeatable and high fidelity measures of the system one seeks to represent, not symptoms or biomarkers. For the first time in LVSD-HF, this novel work enables both parties to use data that is academically rigorous, clinically meaningful and enable the creation of patient and disease specific models. Depending on the stage of the LVDS-HF, the modellers can choose the variables to fit their theoretical cohort.

Previously, it was felt that the LV afterload, comprising total peripheral resistance and systemic vascular compliance had an important role in modelling the LV performance accurately. However, this body of work, comprising the largest cohort of LVSD-HF models based on real patients to date, demonstrates there is no significant different in compliance or resistance between even healthy normals and end-stage. This is important as it demonstrates, that in LVSD-HF and in this this model at least, the most important factor is Emax or peak contractility of the LV. Of course, in reality, it is well known that increased afterload on the heart, due to diseases like hypertension, leads to increase LV wall shear stress, left ventricular hypertrophy and if untreated LVSD and eventually LVSD-HF. Afterload and compliance will also be influenced by other disease processes and medications, which were impossible to control for and very few of this patient cohort, particularly in stage A had hypertension. Our findings however, further strengthen the importance of using load-independent, repeatable and robust measures of LV performance such as elastance when modelling LVSD-HF.

There is wide distribution of the PV loops within each stage, as demonstrated in Stage D by figure 6 (other stages are similar, but for brevity they were not shown), which means that there is difficulty stratifying the disease condition based on current measures of LV function, such as LVEDV or SV. The mean loops from stages B-C would also fit within the data range of stage D and the AHA/ACC classification. This probably due to the lack of standardisation of LV volume against body surface area e.g ml/m^2^ as undoubtedly in a cohort of over 200 patients, there will be large variations in patient size and anatomy. Hence, there is overlap amongst the disease stages and accounts for the non-significance difference in LV volume between the 5 stages. This further highlights the importance of objective differentiation of patient LVSD-HF stages, when creating models, rather than being based on the patient's subjective assessment (although the latter is clinically important) and objective echocardiographic measurements used in current clinical practice.

### Future work

It would be interesting to compare the PV loops of patients with HF of different aetiologies, such as ischaemic versus idiopathic DCM, and to model the effects of various therapies on the different parameters, such as cardiac resynchronisation therapy (CRT). Furthermore, comparing those PV loops of patients with different isolated risk factors for HF, such as obesity or essential hypertension or with differing structural heart diseases such as asymptomatic aortic stenosis or left ventricular hypertrophy to see how they progress from symptomless risk to symptomatic HF would also be of interest. Unfortunately, such data are not currently available. More PV loops are needed to inform stages O and B, as the mean loops and therefore the models, are based on a small number of cases. However, it is unlikely that ethical approval for invasive PV loop analysis would be granted for healthy subjects.

### Drawbacks

Most of the information given about patients was not specific to the loop and so we were unable to control for age, body mass, sex, medications and other relevant co-morbidities (both cardiac, such as mitral regurgitation and non-cardiac, such as renal failure), which could have an impact on the loop shape, size and position [42]. Some curves were averaged already from a patient population (hence the number of patients represented is greater than the number of loops) and what may have been AHA D in previous decades, may be considered AHA C today. We could not control for any differences in methodology across the papers. Most of the PV loops are from middle-aged, probably white, male patients, reflecting the fact the women are under-represented in HF clinical trials. We should not assume women, ethnic minorities, or patients with conditions not represented e.g. obesity, will be the same. As can be seen from figure 6, averaging the means that a lot of the raw data is lost and the curves smoothed accordingly, thus a real patient in stage D will not necessarily meet the mean values, but may fall within the range of values given. Digitizing the PV loops was employed to allow access to the data underlying each loop. Due to the duration of time that has passed since the original publication of many of the papers, only a small number of authors were contactable.

### Conclusions

For the first time, the authors have created a visual and quantitative representation of the AHA/ACC stages of LVSD-HF, from normal to end-stage. The study demonstrates that robust, load-independent and reproducible parameters, such as elastance, can be used to categorise and model HF, complementing the existing classification. The modelled PV loops establish previously unknown physiological parameters for each AHA/ACC stage of LVSD-HF, such as LV elastance and highlight that it this parameter alone, in lumped parameter models, that determines the severity of HF. This is the largest collection of LV PV loop data, which has been used to create stage specific HF models, and as such, will enable cardiovascular modellers to create more accurate models of the heart as it fails and should be used as a reference for future work in this field.

## Supporting Information

Checklist S1 **Search yield according to the Preferred Reporting Items for Systematic Reviews and Meta-Analyses.**
(DOC)

Diagram S1 **Flow diagram for search results according to the Preferred Reporting Items for Systematic Reviews and Meta-Analyses.**
(DOC)
