# Supplementary material for: Closing the Loop: Modelling of Heart Failure Progression from Health to End-Stage Using a Meta-Analysis of Left Ventricular Pressure-Volume Loops
Source: PLoS One. 2014 Dec 5;9(12):e114153. doi: 10.1371/journal.pone.0114153 (PMC4257583; doi:10.1371/journal.pone.0114153)
Supplement: Diagram S1 — Flow diagram for search results according to the Preferred Reporting Items for Systematic Reviews and Meta-Analyses. (DOC) [file pone.0114153.s002.doc]

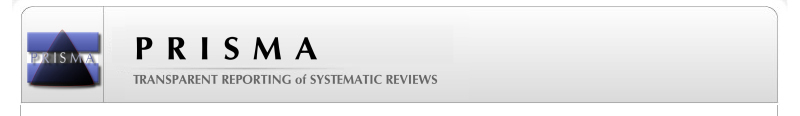
**PRISMA 2009 Flow Diagram**

**Screening**

**Included**

**Eligibility**

**Identification**

Records identified through database searching
(n = 300)

Additional records identified through other sources
(n = 51)

Records after duplicates removed
(n = 260)

Records screened
(n = 97)

Records excluded
(n = 66)

Full-text articles assessed for eligibility
(n = 31)

Full-text articles excluded, with reasons
(n = 0 )

Studies included in qualitative synthesis
(n = 31)

Studies included in quantitative synthesis (meta-analysis)
(n = 31)
